# Prevalence of Articular Surface Injuries in Patients Undergoing Meniscal Surgery: A Retrospective Analysis of 758 Cases

**DOI:** 10.7759/cureus.66789

**Published:** 2024-08-13

**Authors:** Juanjose Valderrama, Xabier Carredano, Agustín León, Cristóbal Vigueras, Felipe Marín, Marcelo Acevedo, Rodrigo Hernández, Gunther Redenz

**Affiliations:** 1 Department of Orthopedics, Clínica Indisa, Santiago, CHL; 2 Department of Orthopedics, Hospital Clínico Mutual de Seguridad, Santiago, CHL; 3 Department of Orthopedics, Hospital Clínico Dra. Eloísa Díaz Insunza de La Florida, Santiago, CHL; 4 Department of Medicine, Universidad Andrés Bello, Santiago, CHL

**Keywords:** meniscal tear, degenerative tears, arthroscopy knee, chondral lesions of knee, meniscal tear pattern

## Abstract

Background and aim

Meniscal tears are often associated with articular surface damage, which could be an important factor in the clinical outcome. However, these concomitant lesions are usually reported as binary variables. Reports of the severity/extent of the concomitant lesions and stratification by meniscal tear are scarce in the literature; in addition, sample sizes of previous reports are limited. This study aimed to characterize meniscal lesions, determine the prevalence of articular surface lesions and their severity, and correlate these lesions with meniscal injury characteristics.

Methods

A cross-sectional study of patients undergoing meniscal surgery between 2017 and 2023 was conducted. Patient characteristics and arthroscopic findings on the location and type of meniscal injury as well as the degree of chondral lesion (sICRS score) were recorded by the surgeon. Statistical analysis included frequency reporting for patient characteristics and study variables, including the median and interquartile range of the sICRS classification of articular surface lesions. Meniscal tear types were categorized as degenerative or non-degenerative to explore associations with chondral injury. Chi-square test and univariate and multivariate logistic regression models were employed to analyze relationships between variables.

Results

A total of 758 surgeries were analyzed, with a mean age of 39.56 years (SD: 12.71) and 67.90% male participants. Medial meniscus injuries accounted for 57.52%, lateral meniscus 36.02%, and both menisci 6.64%. Significant differences were found in vascular area, topography, and lesion type between isolated medial and lateral meniscus lesions (p<0.01). Chondral lesions were present in 35.22% of cases, with significant differences among meniscal injury types (p<0.01). Degenerative tears showed higher rates of chondral damage compared to non-degenerative tears, particularly in lateral meniscus injuries (p<0.01). Regression analysis identified age, gender, meniscal injury characteristics, and meniscectomy percentage as risk factors for articular surface injuries.

Conclusion

Articular surface injuries frequently accompany meniscal lesions, with associations between affected menisci and articular damage extent. Femoral condyles show greater involvement corresponding to compartment-specific meniscal lesions, unlike tibial plateaus. Meniscal degeneration is present in about half of articular cartilage injury cases. Some meniscal tear types may relate to more severe articular lesions, but larger studies are needed to confirm these findings and explore other tear patterns.

## Introduction

Arthroscopic knee surgery due to meniscal pathology is one of the most common procedures [1]. The incidence of meniscal tears increases with age, until around 60 years, and is expected to continue rising [2]. Generally, it presents with highly variable injury patterns [3], which are associated with traumatic events in young patients and degenerative changes in adults [4].

Articular cartilage lesions are commonly encountered during arthroscopy [5], particularly when a concomitant anterior cruciate ligament injury is present [6-8]. According to Fodor et al., the prevalence of articular surface injuries is found to be 50-60% in the medial femoral condyle and 10-15% in patella, with both areas being the most affected [9]. These chondral lesions in the knee may cause premature osteoarthrosis, and result in impaired joint function and decreased quality of life [9,10].

In particular, complex/multidirectional meniscal tears are associated with a higher incidence and severity of cartilage degeneration when compared to other meniscal tears [11]. In addition, it has been reported that cartilage injuries of intermediate severity (Outerbridge grades II and III) present poor functional performance, whereas grade IV injuries usually require future surgery [12].

Meniscal tear patterns and concomitant chondral damage could be important predictors for the post-operative evolution of these injuries. However, there is little high-level evidence exploring this stratification as these are usually reported as binary variables (present/absent) [13]. In addition, the type and severity of chondral injury are not usually reported; thus, the association has not been thoroughly studied [6-8]. Recognizing the interaction between these variables is critical for planning future clinical trials for the efficacy evaluation of different interventions. This study aimed to (i) characterize meniscal lesions, (ii) determine the prevalence of articular surface lesions and their severity, and (iii) correlate these lesions with meniscal injury characteristics.

## Materials and methods

Study design and data source

This cross-sectional prevalence study was conducted and reported according to the Strengthening the Reporting of Observational Studies in Epidemiology (STROBE) guidelines. A database generated by a team of knee surgeons in a sports health center was retrospectively analyzed. The database contained a registry of consecutive meniscal surgeries and was collected between April 2017 and December 2023.

Data collection and eligibility criteria

Protocols for ligament reconstruction surgery, type of meniscal injury and its treatment, type of chondral injury, sex, and age were recorded by the treating surgeon. As primary variables, evaluated through arthroscopic observation, the location and type of meniscal injury [14], as well as the degree of chondral injury according to the International Cartilage Repair Society (ICRS), without subgroups, are as follows: grade 0 indicates the absence of lesion; grade I represents superficial lesions, such as chondromalacia, superficial fissures, and soft indentations; grade II includes lesions that extending to less than 50% of the cartilage depth; grade III indicates cartilage defects that extend beyond 50% of the cartilage depth which may also involve the calcified layer, but not the subchondral bone; and grade IV, lesions that extend through the subchondral bone [15]. Observations of patients undergoing meniscal surgery whose surgical records describe the meniscal tear type, location, or treatment were included. Anterior cruciate ligament surgeries were excluded.

Statistical analysis

Patient characteristics and variables under study, including meniscal and chondral damage, are reported as frequencies. The degree of chondral damage (sICRS) is described by the median (p25-p75). Differences between medial and lateral meniscus and the influence of the affected meniscus on the prevalence of articular surface damage were tested using the chi-square test of independence. The association between the type of meniscal tear and the prevalence of chondral injury was explored by grouping the meniscal tear type into degenerative and non-degenerative.

In addition, this association was explored with several univariate logistic regressions using the articular surface lesion as the response variable. Variables found to be significant in this model were incorporated into a multivariate model. All analyses were performed using Stata 15 software (College Station, TX: StataCorp LLC), and the significance level was determined using an alpha <0.05.

Ethics approval

A secondary database was analyzed. The objectives of this study did not require patient identifiers, and the investigators had access to a limited dataset. The protocol for anonymization and data access was approved by the Ethical Committee of the same center (authorization number: 0012021).

## Results

A total of 758 observations were analyzed. The mean age of the sample was 39.56 years (SD: 12.71 years), and 67.90% were males. A total of 57.52% corresponded to medial meniscus injuries, 36.02% to lateral meniscus, and 6.64% to both menisci. Approximately 92% of the patients underwent partial meniscectomy, with an average resection rate of 29.59%. The four surgeons performing the most surgeries accumulated 87.04% of the registries.

Tear characteristics, topography, and area involved are shown in Table 1. When comparing isolated medial and lateral meniscus lesions, differences in vascular area, topography, and lesion type were found (chi-square test: p<0.01). The median meniscectomy rate was 25% (p25-p75: 15-35%), with no differences between the medial, lateral, or both menisci groups.

**Table 1:** Tear characteristics - topography, area, and type. *P-value of <0.01 was considered statistically significant in the chi-square test of independence. WW: white-white; RW: red-white; RR: red-red; PH: posterior horn; AH: anterior horn

Variables		Isolated tear		Both menisci injured (n=49)	
		Medial meniscus (n=432), n (%)	Lateral meniscus (n=270), n (%)	Medial meniscus, n (%)	Lateral meniscus, n (%)
Tear zone*	WW	36 (8.33)	81 (30.00)	9 (18.75)	29 (59.18)
	RW	350 (81.02)	166 (61.48)	30 (62.50)	15 (30.61)
	RR	46 (10.65)	23 (8.52)	9 (18.75)	5 (10.20)
Topography*	Body	13 (2.98)	96 (35.29)	5 (10.42)	24 (48.98)
	PH	167 (38.3)	6 (2.21)	17 (35.83)	7 (14.29)
	Body-PH	198 (45.41)	61 (22.43)	22 (45.83)	7 (14.29)
	AH	2 (0.46)	15 (5.51)	-	2 (4.08)
	Body-AH	2 (0.46)	42 (15.44)	-	4 (8.16)
	AH-body-PH	53 (12.16)	51 (18.75)	4 (8.33)	5 (10.20)
	AH-PH	1 (0.23)	1 (0.37)	-	-
Tear type*	Degenerative	182 (42.03)	51 (18.89)	29 (59.18)	18 (36.73)
	Bucket handle	50 (11.55)	19 (7.04)	5 (10.20)	2 (4.08)
	Multidirectional	59 (13.63)	49 (18.15)	3 (6.12)	4 (8.16)
	Radial	55 (13.70)	80 (29.63)	1 (2.04)	17 (34.69)
	Longitudinal	45 (10.39)	30 (11.11)	9 (18.37)	3 (6.12)
	Horizontal	11 (2.54)	16 (5.92)	-	2 (4.08)
	Flap	28 (6.47)	23 (8.52)	1 (2.04)	3 (6.12)
	Disinsertion	2 (0.46)	1 (0.37)	1 (2.04)	-
	Ramp	1 (0.23)	-	-	-
	Post-meniscectomy	-	1 (0.37)	-	-

There were 267 (35.22%) records of one or more lesions on articular surfaces (sICRS classification ≥1). A total of 47.57% of injured cases presented only one affected surface. The prevalence of these lesions was 39.22% in patients treated for medial meniscus injury, 26.37% for lateral meniscus injury, and 48.98% in those treated for both menisci, this difference was statistically significant (chi-square test: p<0.01). The presence of chondral injury on the different articular surfaces and the degree of injury according to the sICRS classification for isolated meniscal lesions are shown in Table 2.

**Table 2:** Associated articular surface lesions in isolated meniscal tears. *P-value of <0.01 was considered statistically significant in the chi-square test of independence. The presence of articular surface lesions is not exclusive.

Variables	Medial meniscus (n=235)		Lateral meniscus (n=143)	
	n (%)	sICRS median (p25-p75)	n (%)	sICRS median (p25-p75)
Lateral femoral condyle*	34 (7.80)	2 (1-3)	33 (12.09)	3 (2-3)
Medial femoral condyle*	91 (20.87)	3 (2-3)	14 (5.13)	3 (3-3)
Lateral tibial plateau*	75 (17.20)	2 (1-3)	45 (16.48)	2 (2-3)
Medial tibial plateau*	66 (15.14)	2 (1-2)	10 (3.66)	1.5 (1-2)
Patella*	85 (19.50)	2 (2-3)	17 (6.23)	2 (1-3)
Trochlea*	29 (6.65)	3 (2-3)	8 (2.93)	2 (1.5-3.5)

The sample was grouped into degenerative and non-degenerative tears based on the injury pathophysiology. The association between the presence of chondral damage and degenerative changes in the meniscus was thus explored. Articular surface lesions were observed in 22.07% and 45.10% (chi-square test: p<0.01) of non-degenerative and degenerative lateral meniscal lesions, respectively. Articular surface lesions were observed in 35.83% and 43.96% (chi-square test: p=0.09) of non-degenerative and degenerative medial meniscus lesions, respectively.

Exploratory regression analysis showed that tear zone and topography are not associated with articular surface injuries. On the other hand, age, gender, meniscal injury, tear type, and percentage of meniscectomy are risk factors. Particularly, degenerative, radial, and bucket handle tears were a risk factor for these concomitant lesions. Table 3 presents the complete statistical results of this regression analysis including odds ratios and confidence intervals for each factor.

**Table 3:** Univariate and multivariate logistic regression analysis of articular surface injuries. Ref refers to the regression constant or level of reference. Tear-type variables were not used in the multivariate analysis as these are mutually exclusive in the database.

Variables	Categories	Univariate regression OR (95% CI)	Multivariate regression OR (95% CI)
Age	>54 years	4.74 (3.03-7.42)	4.42 (2.76-7.07)
	54 years or less (Ref)	-	-
Gender	Female	1.76 (1.28-2.41)	1.69 (1.21-2.36)
	Male (Ref)	-	-
Meniscal injury	Medial meniscus	1.80 (1.29-2.51)	1.50 (1.05-2.13)
	Both menisci	2.68 (1.44-4.99)	1.94 (1.00-3.38)
	Lateral meniscus (Ref)	-	-
Medial meniscus: tear type	Bucket handle	1.61 (0.72-3.60)	-
	Disinsertion	1.30 (0.11-15.41)	-
	Horizontal	2.60 (0.58-11.75)	-
	Degenerative	2.21 (1.15-4.26)	-
	Multidirectional	0.83 (0.36-1.91)	-
	Flap	1.36 (0.52-3.61)	-
	Radial	2.79 (1.26-6.17)	-
	Longitudinal (Ref)	-	-
Lateral meniscus: tear type	Bucket handle	4.95 (1.45-16.96)	-
	Disinsertion	-	-
	Horizontal	3.21 (0.75-13.69)	-
	Degenerative	4.63 (1.70-12.63)	-
	Multidirectional	1.78 (0.61-5.17)	-
	Flap	1.66 (0.48-5.72)	-
	Radial	0.70 (0.24-2.01)	-
	Longitudinal (Ref)	-	-
Meniscectomy percentage	-	1.01 (1.01-1.02)	1.02 (1.01-1.02)

## Discussion

This study aimed to characterize articular surface lesions in meniscal injury patients undergoing arthroscopy by correlating the site of injury with the characteristics of the meniscal tear. We found a prevalence of chondral lesions observed by arthroscopy of 35.22%, which would be more frequent in patients with injuries in both menisci (48.98%).

This prevalence is below the 60-70% reported in studies evaluating consecutive arthroscopy for various causes [9]. Although there is some consensus in the literature regarding the prevalence of these lesions, some reports have identified a prevalence of approximately 50% [16]. These differences could be determined by different patient selection criteria; for example, the inclusion of patients with anterior cruciate ligament injury, which increases the risk of articular cartilage injury [17].

Regarding the description of meniscal lesions, these tend to occur in the posterior horn [18,19]. When analyzing the location according to the injured meniscus, we found a significant prevalence of tears in the lateral meniscus body (35.29%), so the injury pattern is more centralized in this meniscus [20]. Furthermore, up to 20% of patients with lateral meniscus injury had the anterior horn affected in some way.

When analyzing the characteristics of medial meniscus injuries, previous reports indicated these lesions would not occur in the red-red zone, possibly because of the limited number of patients included in those samples [17,21]. Red-red zone lesions in the medial meniscus were found to be rare; however, they could occur in approximately 4% of patients. In general, lesion characteristics will vary when comparing the medial and lateral meniscus, so we suggest analyzing by subgroups according to the affected meniscus.

The prevalence of articular surface damage and its relationship with meniscal degeneration was explored. According to our results, articular surface injuries could occur independently of the presence of a degenerative process in the medial meniscus (chi-square test: p=0.10). As indicated by Beaufils and Pujol, a degenerative process is more likely to occur in the medial compartment [22].

Previous reports, using a similar methodology to ours when reporting articular surface damage show some agreement that, in the presence of medial meniscus lesions, there is a higher probability of presenting lesions of the articular surface of the medial compartment and patella [9,16,21]. An exception to this rule can be seen in the investigation of Spahn et al., presumably due to the sample size [21].

While the degree of injury according to the sICRS grade observed is similar to that reported by Spahn et al., there are differences when analyzing according to the affected meniscus [21]. This series represents a five-fold increase in sample size compared to that previous reference, allowing a more precise estimation. The lateral femoral condyle appears to exhibit a significant degree of injury when a lateral meniscal tear is present, while damage to the tibial surfaces is not as important. The high degree of injury to the trochlea is noteworthy; however, as in the previous study, the sample size was small, and this observation may be due to other factors such as patellofemoral pathology.

Risk factors for articular cartilage deterioration have been previously studied. Increased meniscal resection, overweight, genetic predisposition, and female sex are associated with radiological evidence of osteoarthrosis [12,23]. It has been postulated that bucket-handle lesions are not associated with chondral lesions; however, our exploratory analysis showed that they are an important risk factor in lateral meniscal tears [11].

Limitations to this study include the lack of relevant variables that were not recorded in the database, such as the mechanism of injury or limb axis, as an increase in degenerative processes in the medial compartment in the presence of varus has been reported [24]. Despite having a larger number of cases than previous studies, the results still lack a robust estimator in some cases. In addition, a multicenter effort is required to increase the external validity of the results.

## Conclusions

Injuries to the articular surface in patients undergoing meniscal surgery are highly prevalent. The affected meniscus has an important association with the area and severity of the articular surface lesion. In general, the femoral condyles are more affected in association with their respective meniscal lesions according to the compartment, but not the tibial plates.

A degenerative process of the meniscus is present in about half of the cases of individuals with articular cartilage injuries. A larger volume of observation in future studies is required to analyze the association with other types of meniscal tears. In our exploratory analysis, in contrast to some studies, bucket-handle, radial, and degenerative lesions could be related to major articular surface lesions.
